# Literary Notes

**Published:** 1899-03

**Authors:** 


### LITERARY NOTES.

---

THE *International Medical Magazine*, heretofore published in Philadelphia, will henceforth be published in New York, by E. B. Treat & Co., 241-243 West 23d street. It will, however, continue to be edited by Dr. Boardman Reed, of Philadelphia, to whom, at 1928 Chestnut Street, all literary communications and exchanges should be addressed.

---

THE *Ohio Medical Journal* was discontinued after the publication of the edition for December, 1898.

---

THE *North American Practitioner* (Chicago) appeared in a new dress and in improved form with the beginning of 1899. The prosperity of this excellent magazine is indicated by its appearance and valuable contents.

---

THE *Western Clinical Recorder* (Chicago) made its first appearance in January, 1899. Its initial number is handsomely printed and it contains excellent material. It is conducted by Drs. Fred. Jennings Hodges and William T. Rinehart.

OBSTETRICS is the name of a new monthly journal that is edited by Dr. Edward A. Ayers, professor of obstetrics in the New York Polyclinic. It is published by the Van Publishing Company, New York, and its subscription price is $2.00 a year.

